# Cytosolic DNA crosstalk in senescence: a new axis of inflammatory signaling?

**DOI:** 10.1038/s44318-025-00531-z

**Published:** 2025-08-29

**Authors:** Zhixun Dou, Jill A Kreiling, Susanne Heynen-Genel, Diana Jurk, Nicola Neretti, Peter D Adams, John M Sedivy, João F Passos

**Affiliations:** 1 Krantz Family Center for Cancer Research, Massachusetts General Hospital, Boston, MA USA; 2 Harvard Stem Cell Institute, Harvard University, Cambridge, MA USA; 3 Department of Medicine, Massachusetts General Hospital, Harvard Medical School, Boston, MA USA; 4 Center on the Biology of Aging, and the Department of Molecular Biology, Cell Biology and Biochemistry, Brown University, 225 Dyer Street, Providence, RI 02903 USA; 5 Sanford Burnham Prebys Medical Discovery Institute, La Jolla, CA 92037 USA; 6 Robert and Arlene Kogod Center on Aging and the Department of Physiology and Biomedical Engineering, Mayo Clinic, Rochester, MN 55905 USA

**Keywords:** Cell Cycle, Immunology, Molecular Biology of Disease

## Abstract

This comment explores potential synergies of accumulating mitochondrial DNA, chromatin fragments, and LINE-1 retrotransposon-derived cDNAs in senescence and chronic inflammation.

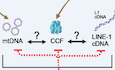

## Teaser

Cellular senescence is a form of stable growth arrest that contributes to aging and age-related diseases, in part through the senescence-associated secretory phenotype (SASP). Recent studies show that senescent cells accumulate several species of cytosolic DNAs, including mitochondrial DNA (mtDNA), cytoplasmic chromatin fragments (CCFs), and retrotransposable element cDNAs, which collectively activate the cGAS–STING pathway and drive SASP expression. Surprisingly, downregulating any one of these DNA species is often enough to suppress the SASP, raising key questions about their functional interactions. We propose that these cytosolic DNA species do not act in isolation but instead either follow a coordinated sequence or engage in synergistic crosstalk to amplify and sustain inflammatory signaling. While therapeutic approaches directly targeting the cGAS–STING pathway are being developed, we argue that blocking the sources of cytosolic DNA might be a more specific and safer strategy to target the deleterious effects of senescent cells. In particular, this approach should enable reducing chronic inflammation without impairing important immune functions, offering a new direction for therapies aimed at promoting healthy aging.

Cellular senescence is an irreversible cell cycle arrest that occurs in response to a range of intrinsic and extrinsic stressors, including telomere attrition, DNA damage, oxidative stress, and oncogene activation. While senescence serves as a critical tumor-suppressive and wound-healing mechanism, its persistence in tissues can become deleterious with age (Muñoz-Espín and Serrano, 2014). A hallmark feature of senescent cells is the acquisition of a complex proinflammatory secretome, termed the senescence-associated secretory phenotype (SASP). The SASP is comprised of a wide array of cytokines, chemokines, proteases, and growth factors that can remodel the tissue microenvironment, reinforce senescence in neighboring cells, and drive chronic sterile inflammation (Coppé et al, 2010).

Senescent cells accumulate in various tissues with aging, and they are also found in numerous age-related pathologies, including osteoarthritis, atherosclerosis, pulmonary fibrosis, and neurodegeneration. Their accumulation contributes to tissue dysfunction, stem cell exhaustion, and immune dysregulation. Importantly, the selective elimination of senescent cells in preclinical models improves multiple aging phenotypes and extends healthspan, establishing senescence as a therapeutic target. Consequently, substantial effort has been directed at developing senolytics (agents that selectively kill senescent cells) and senomorphics (agents that suppress the SASP without killing the cell) as strategies to mitigate age-related diseases (Pignolo et al, 2020; Wang et al, 2025).

Recent publications have uncovered a central role for cytosolic DNA sensing in mediating the SASP in senescent cells. Specifically, senescent cells have been shown to accumulate multiple distinct types of cytosolic DNA, including mtDNA, cytoplasmic chromatin fragments (CCFs) and retrotransposon-derived cDNA (Miller et al, 2021).

These DNA species have been demonstrated to activate the cytosolic DNA sensor cyclic GMP-AMP synthase (cGAS), which in turn activates the STING (stimulator of interferon genes) pathway. STING recruits and activates TBK1, leading to phosphorylation of IRF3 and induction of interferon-stimulated genes (ISGs). STING signaling has also been associated with activation of NF-κB, although the precise mechanisms remain incompletely defined. Together, IRF3 and NF-κB orchestrate the expression of the SASP. Thus, cGAS–STING signaling provides a direct mechanistic link between cytosolic DNA and the inflammatory phenotype of senescent cells.

However, an unresolved paradox is apparent: Interventions that target any one of these DNA species individually, whether mtDNA, CCFs, or retrotransposon cDNA, have each been proposed to markedly suppress the SASP, both in vitro and in vivo. This is surprising, given that the other cytosolic DNA species are presumed to remain present and, in principle, could still activate cGAS–STING signaling. It remains unclear whether these interventions are truly specific or whether they affect multiple cytosolic DNA species simultaneously.

These observations raise several fundamental questions that are currently unanswered:

- How can the inhibition of a single cytosolic DNA species suppress the SASP if the others remain intact and active?
- Alternatively, does suppression of one species lead to a coordinated reduction in the abundance of the others?
- Or perhaps, are the different cytosolic DNA species interdependent in their signaling capacity, such that one acts as a master regulator or permissive factor for the others?

**Figure 1:**
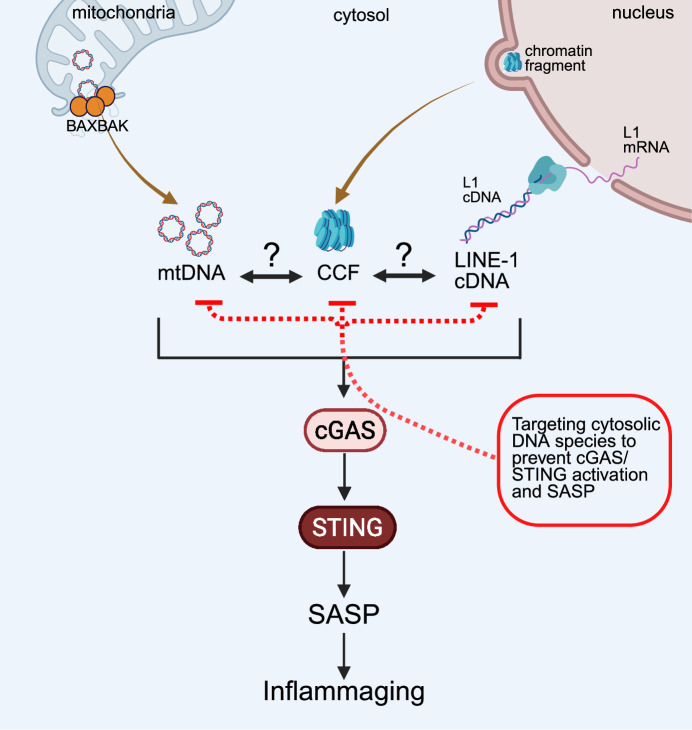
mtDNA, CCFs, and LINE-1 cDNA accumulate in the cytosol of senescent cells and activate the cGAS–STING pathway, leading to SASP expression and inflammaging. The relative contributions and potential crosstalk between these DNA species remain unknown. Further research is needed to define their origins, regulation, and individual inflammatory consequences. We propose that targeting cytosolic DNA species to prevent cGAS–STING activation represents a promising therapeutic strategy to mitigate inflammaging.

These questions point to an unappreciated level of crosstalk and cooperation among cytosolic DNA species in senescent cells. Rather than functioning as independent, redundant activators of cGAS–STING, mtDNA, CCFs, and retrotransposon cDNA may form a coordinated and possibly self-reinforcing network (Fig. 1). This concept represents a significant shift in our understanding of SASP signaling in senescence and opens new avenues for therapeutic intervention.

In this perspective, we explore the emerging landscape of cytosolic DNA and interplay in senescence, propose conceptual models to explain their cooperative behavior, and discuss the potential of targeting the upstream DNA sources, rather than the shared cytosolic DNA sensing machinery, as a more precise and potentially safer strategy to blunt chronic inflammation and promote healthy aging.

## Different cytosolic DNA species in senescent cells drive the SASP

Multiple types of cytosolic DNA contribute to the activation of the SASP. One key source is mitochondria. Senescent cells display mitochondrial dysfunction, including altered dynamics, loss of membrane potential, increased reactive oxygen species (ROS), and reduced mitophagy (Martini and Passos, 2023). One mechanism by which mitochondria contribute to SASP is through sublethal minority mitochondrial outer membrane permeabilization (miMOMP), which allows mtDNA to leak into the cytosol and activate the cGAS–STING pathway (Riley et al, 2018; Victorelli et al, 2023). In addition, recent data implicate the voltage-dependent anion channel (VDAC) in contributing to cytosolic mtDNA accumulation during senescence and aging (Gulen et al, 2023; Lai et al, 2025).

In addition to mtDNA, nuclear-derived DNA also plays a major role in driving the SASP. Senescent cells accumulate cytoplasmic chromatin fragments (CCFs), which form through nuclear membrane blebbing and egress after cell-cycle arrest (Kumazawa et al, 2024). CCFs are associated with persistent DNA damage and impaired repair, common features of senescence (Miller et al, 2025). Once in the cytoplasm, CCFs can activate the cGAS–STING pathway and promote SASP expression (Dou et al, 2017). These fragments contain chromatin proteins and DNA damage markers such as γH2A.X (Ivanov et al, 2013). CCFs may also be targeted by nuclear autophagy (Dou et al, 2015), but their ultimate fate, whether degraded, secreted, or involved in alternative signaling pathways, remains poorly understood. Importantly, CCFs are not only observed in vitro but also detected in vivo, such as in the livers of mice undergoing irradiation, oncogene- or acetaminophen-induced senescence (Dou et al, 2017; Vizioli et al, 2020).

A third source of cytosolic DNA comes from retrotransposons (Gorbunova et al, 2021), which are transcriptionally upregulated in senescent cells (De Cecco et al, 2019; Liu et al, 2023) due to epigenetic changes and heterochromatin relaxation. Mammalian genomes contain multiple retrotransposon clades, including endogenous retroviruses (ERVs), long interspersed nuclear elements (LINEs) and short interspersed nuclear elements (SINEs). Genetic and pharmacological evidence points to reverse transcribed LINEs as an important source of cytosolic DNA (De Cecco et al, 2019; Fukuda et al, 2021; Simon et al, 2019). Normally, these elements transpose through a nuclear reverse transcription process, but in senescent cells, the cDNAs accumulate in the cytoplasm (Baldwin et al, 2024). Like mtDNA and CCFs, these cytoplasmic cDNAs activate cGAS–STING signaling, leading to type I interferon (IFN-I) responses and SASP. Notably, reverse transcriptase inhibitors, such as lamivudine, block cDNA synthesis and reduce both IFN-I signaling and SASP expression (De Cecco et al, 2019). Together, these findings highlight that senescent cells harbor multiple sources of cytosolic DNA that converge on the cGAS–STING pathway to the SASP.

## How can multiple cytosolic DNA species drive the SASP?

One of the most intriguing and puzzling findings in the field is that inhibiting just one type of cytosolic DNA species, whether mtDNA, CCF, or retrotransposon-derived cDNA, is often enough to suppress the SASP. But if the other DNA species are still present and potentially active, how is the SASP still silenced? To explain this paradox, we propose two distinct, but not mutually exclusive, mechanistic hypotheses that remain to be tested.

The first hypothesis is that different cytosolic DNA species accumulate in a manner that is dependent on each other, perhaps in a temporal sequence during the establishment of senescence. For example, mtDNA leakage may occur early, followed by CCF formation, with LINE-1 cDNA appearing later. In this model, disrupting an early event, such as mtDNA release, could prevent or blunt the downstream accumulation of other DNA species, thereby stopping the full activation of the SASP cascade. Consistent with this idea, mitophagy-mediated depletion of mitochondria from senescent cells prevents the release of mtDNA into the cytoplasm (Victorelli et al, 2023) and also the accumulation of CCF (Vizioli et al, 2020). Published data hint that LINE-1 elements are activated later in the senescence process (De Cecco et al, 2019), but we currently lack a precise timeline for the formation of cytosolic mtDNA, CCFs and LINE-1 in the same cell type under the same conditions. Addressing this will require assessment of each of the cytosolic DNA species after targeted inhibition of just one species and carefully designed time-course experiments, tracking the dynamics of each DNA species alongside key SASP markers during senescence induction.

The second hypothesis is that these cytosolic DNA species function in a cooperative or synergistic manner. That is, no single species may be sufficient on its own to sustain full cGAS–STING activation and drive the robust inflammatory signaling seen in senescence. Instead, the combined presence of multiple DNA species could amplify the magnitude or duration of signaling, ensuring a strong and persistent SASP. Testing this will require evaluation of cGAS/STING activation and expression of SASP after only one of the cytosolic DNA species is selectively removed, as well as combinatorial inhibition of mtDNA release, CCF formation, and LINE-1 reverse transcription.

Importantly, these mechanisms may vary depending on the cell type and the triggering senescence stimulus, factors that could influence not only which DNA species accumulate, but also how they interact. While we focus our discussion primarily on the cGAS–STING pathway for simplicity, we acknowledge that other cytosolic DNA-sensing mechanisms, as well as RNA-sensing pathways, may also contribute to SASP regulation and merit further investigation. Together, these hypotheses offer a roadmap for dissecting the complex and layered nature of cytosolic DNA signaling in senescence, with implications for designing more effective SASP-targeted interventions.

## Why should we target cytosolic DNA, instead of the downstream signaling?

Given the complexity and diversity of cytosolic DNA species in senescent cells, one might argue that it would be more straightforward to target the shared DNA-sensing pathways, such as cGAS–STING, rather than the cytosolic DNA itself. However, we propose that targeting the source of cytosolic DNA offers distinct and potentially superior advantages.

Data from the authors of this perspective (as well as others) demonstrate that inhibiting the accumulation of cytosolic DNA, whether mtDNA, CCF, and/or LINE-1-derived cDNA, can suppress SASP expression, reduce age-related inflammation, and improve healthspan outcomes in mice and humans (De Cecco et al, 2019; Dou et al, 2017; Magagnoli et al, 2025; Simon et al, 2019; Victorelli et al, 2023). For example, MDM2 inhibitors that block accumulation of CCF, and potentially other cytosolic DNAs (see above), suppress SASP and immune infiltration and restore metabolic gene expression in aged liver (Miller et al, 2025). Inhibition of mtDNA release using BAX inhibitor (BAI1) suppresses inflammation and frailty in aged mice (Victorelli et al, 2023). In aged mice, lamivudine treatment alleviates systemic inflammation and improves multiple aging-related pathologies, including cognitive decline (Wahl et al, 2023). Human studies have further linked antiretroviral therapy to reduced incidence of age-associated diseases (Magagnoli et al, 2025) and even deceleration of epigenetic aging (Sehgal et al, 2024), highlighting retrotransposon inhibition as a promising strategy to promote healthy aging. These findings establish proof-of-concept that cytosolic DNA is not just a byproduct of senescence but a central driver of its inflammatory phenotype and thus represents a valuable therapeutic target.

Importantly, targeting the DNA upstream of the cGAS–STING pathway may avoid the drawbacks of directly inhibiting the pathway itself. While cGAS/STING inhibitors are in development (Barasa et al, 2023; Haag et al, 2018), these approaches carry the risk of broad immunosuppression, which is especially concerning in aging populations already facing immune decline. The cGAS–STING pathway plays essential roles in acute immune responses, including wound healing (Mizutani et al, 2020) and pathogen defense (Jenson and Chen, 2024). Blunting these responses could have unintended and harmful consequences. In contrast, selectively blocking the accumulation of cytosolic DNA may allow us to dampen chronic, sterile inflammation linked to cell senescence, while preserving the beneficial aspects of immune surveillance. In line with this, inhibiting mtDNA release or CCF formation does not affect the acute immune response triggered by cytosolic DNA transfection (Kumazawa et al, 2024; Victorelli et al, 2023).

Taken together, we suggest that the field should look beyond cGAS–STING itself and focus on the diverse sources of cytosolic DNA as the root cause of chronic inflammation in senescence and aging. Targeting these sources may yield more specific, safer, and ultimately more effective interventions to mitigate age-related dysfunction without compromising essential immune functions.

## Supplementary information

Peer Review File
