# Supplementary material for: Cytosolic DNA crosstalk in senescence: a new axis of inflammatory signaling?
Source: EMBO J. 2025 Aug 29;44(19):5239–43. doi: 10.1038/s44318-025-00531-z (PMC12489115; doi:10.1038/s44318-025-00531-z)
Supplement: Supplementary file 1 — Peer Review File [file 44318_2025_531_MOESM1_ESM.pdf]

THE EMBO JOURNAL

Cytosolic DNA crosstalk in senescence: a new axis of inflammatory signaling?

Zhixun Dou, Jill Kreiling, Susanne Heynen-Genel, Diana Jurk, Nicola Neretti, Peter Adams, John Sedivy, and Joao Passos

Corresponding author: Joao Passos (Passos.Joao@mayo.edu)

Review Timeline:

Submission Date:	18th Jul 25
Editorial Decision:	25th Jul 25
Revision Received:	28th Jul 25
Accepted:	29th Jul 25

Editor: Daniel Klimmeck

Transaction Report:

This article was editorially reviewed.
